# Economic insecurity: A socioeconomic determinant of mental health

**DOI:** 10.1016/j.ssmph.2018.09.006

**Published:** 2018-09-15

**Authors:** Daniel Kopasker, Catia Montagna, Keith A. Bender

**Affiliations:** a University of Aberdeen, HERU & CELMR, UK; b University of Aberdeen, CELMR & SIRE, UK

**Keywords:** Economic insecurity, Mental health, Socioeconomic determinants of health

## Abstract

Economic insecurity is an emerging topic that is increasingly relevant to the labour markets of developed economies. This paper uses data from the British Household Panel Survey to assess the causal effect of various aspects of economic insecurity on mental health in the UK. The results support the idea that economic insecurity is an emerging socioeconomic determinant of mental health, although the size of the effect varies across measures of insecurity. In particular, perceived future risks are more damaging to mental health than realised volatility, insecurity is more damaging for men, and the negative effect of insecurity is constant throughout the income distribution. Importantly, these changes in mental health are experienced without future unemployment necessarily occurring.

## Highlights

• Effects of both subjective and objective economic insecurity are analysed within a causal model.
• Fear of unemployment is the most damaging form of economic insecurity, particularly for males.
• The negative effect of insecurity is experienced equally throughout the income distribution.
• Effects observed regardless of unemployment fears being realised.
• Estimates which do not control for simultaneity potentially understate the size of the effect.

## 1 Introduction

Mental health is an issue that is justifiably receiving increasing attention. At the individual level, mental health is often the single biggest contributor to life satisfaction, more so than physical health, unemployment, and income (Layard et al., 2013). Mental health also has substantial economic consequences at the employer and national level. In a report to the Stevenson-Farmer Independent Review of Mental Health and Employers, Hampson et al. (2017) estimate that poor mental health costs businesses around £37bn per year, equivalent to approximately 2% of GDP. The major proportion of these costs result from reduced productivity owing to employees’ mental health.

Using data from the British Household Panel Survey (BHPS) this paper investigates the impact of economic insecurity on mental health within the working-age population of the UK. Through the use of a robust estimation strategy, we identify the most damaging forms of economic insecurity for different sections of the workforce.

Economic insecurity can be defined as “the anxiety produced by the possible exposure to adverse economic events and by the anticipation of the difficulty to recover from them” (Bossert & D’Ambrosio, 2013, p. 1018). Examples could include a fear of unemployment, or an expectation of a worsening financial situation. As employment relationships change, with precarious contract arrangements becoming increasingly normalised, understanding the impact of economic insecurity on population health becomes more important. Arguably the most prominent illustration of changing employment relationships is the recent and increasing use of ‘zero-hours contracts’, which provide employees with no guarantee of income or continuing employment. It is estimated that 2.8% of employees in the UK were employed on zero-hours contacts in their main job in 2017 (ONS, 2018), a figure that has increased substantially over the past decade (Bender & Theodossiou, 2017).

Many studies provide unequivocal evidence of the negative effects of unemployment on mental health (for example, Clark & Oswald, 1994; Theodossiou, 1998; Clark, 2003; Stauder, 2018).^1^ Less attention has been paid to the health effects of a fear of unemployment or expected financial hardship, although Bender and Theodossiou (2017) find that precarious work leads to worse physical health. Clark and Georgellis (2013) suggest there are significant negative effects on mental health prior to the realisation of unemployment expectations. It is possible that this effect may also be experienced by those whose employment remains stable, such that there is a negative impact on mental health regardless of expectations being realised.

This paper identifies a causal impact of economic insecurity on mental health using a verified measure of mental health (Goldberg et al., 1997) – the 12-item General Health Questionnaire (GHQ-12). Our analysis enhances existing models by allowing for the effect of insecurity to vary across a range of key socioeconomic factors. Furthermore, an important limitation within the previous literature is that the direction of causality cannot be reliably determined. It is often assumed that economic insecurity causes a decline in mental health. However, it is straightforward to envisage scenarios within which declining mental health causes changes in economic insecurity. For example, the onset of mental health difficulties may impact on an individual’s performance at work and lead to them being concerned about their future income and employment outcomes. Geishecker (2012) demonstrates that failing to control for this simultaneity bias will result in the negative effect of insecurity on mental health being substantially underestimated, possibly by a factor of between two and three. We address potential simultaneity bias using the incidence of insecurity within individuals’ reference groups as exogenous instruments.

Our analysis suggests that the extent to which the mental health of an individual is affected by economic insecurity varies across measures of insecurity and gender, but not income. For males, the largest effect comes from insecurity related to their employment and the size of this effect is above the widely observed level (Norman et al., 2004) determining a Minimum Detectable Difference (MDD). This level would be considered as a substantial departure from normal functioning by the sufferer and may lead to changes in behaviour, including increased work absenteeism and changes in health behaviours. For females, the effect of work-related economic insecurity and concerns regarding the future financial situation are approximately equal and do not meet the MDD criteria, although the effects remain large in comparison to other determinants of mental health. For both genders it is the case that subjective measures of economic insecurity have a larger negative effect than objective measures, based on realised income volatility, and the size of the effect is not influenced by labour market outcomes.

The MDD criteria approximate a threshold level beyond which the implications of results change. Given this threshold, it becomes increasingly important to be aware of the impact of any bias within estimated coefficients. Consistent with Geishecker (2012), we provide some evidence that models which do not control for endogeneity may be underestimating the effect of economic insecurity on mental health by a factor of two or more. Consequently, the previous literature may understate the importance of economic insecurity to population mental health and the associated productivity implications.

The rest of the paper is organised as follows. Section 2 provides a brief overview of the key related literature. Section 3 covers methodological issues, including the construction of the Economic Security Index (ESI) (Hacker et al., 2014) using the BHPS dataset. The main results are given in Section 4. This section includes a comparison of trends across the measures of economic insecurity, and discussion of the regression results. Section 5 concludes the paper.

## 2 Related literature

A body of empirical evidence has provided robust support for the negative impact on health of exposure to downside economic risks related to employment relationships. The consistency of these findings has led to this form of economic insecurity being identified as an emerging socioeconomic determinant of health (Benach et al., 2014).

Within the existing literature there is evidence that past and future employment experiences influence wellbeing. Clark et al., (2001) illustrates that unemployment experience continues to negatively affect subjective wellbeing despite an individual finding alternative employment. However, using the same data, Knabe and Rätzel (2011) find that this effect is not observed when current concerns regarding job security are controlled for. They suggest that past unemployment experience impacts on current wellbeing by influencing perceptions of future unemployment risk. In addition to finding an effect from past unemployment, Clark and Georgellis (2013) also find a negative effect of future unemployment on both subjective wellbeing and mental health prior to the event being experienced. In each of these papers, clear gender differences are found in the magnitude of the effect. A key distinction between unemployment experience and economic insecurity is that the latter can have a negative impact on health regardless of any objective event occurring.

Using Canadian data, Watson (2015) shows that work-related economic insecurity is associated with a decline in mental health for both males and females. In further sub-group analysis of parents, Watson (2015) finds no statistically significant effect of work-related economic insecurity on mental health for females, while the coefficient for males becomes larger and remains significant. This is not entirely consistent with a “breadwinner hypothesis” since both male and female who are responsible for children should be equally affected by economic insecurity. Watson (2015) posits that a possible explanation is that the “breadwinner” role may be more important to the male identity than to females.

Rohde et al., (2017a) find negative effects on mental health across a range of measures of economic insecurity using data from Australia. The negative effects on mental health are much larger than the impact on physical health. Additionally, mental health effects are not influenced by the income of the sufferer, while a higher income significantly reduces the negative effects on physical health. A further paper from Rohde et al., (2016) finds that a standard deviation shock to a range of economic insecurity variables results in a decline in mental health of approximately 1.4 percentage points.

The existing evidence illustrates the consistency of the finding that economic insecurity has a significant negative effect on mental health. However, these papers do not attempt to address potential simultaneity in the relationship. Although an estimation strategy that controls for time-invariant individual heterogeneity will remove some sources of endogeneity, other time-varying sources may remain since transitory mental health issues may impact on attendance and performance at work. Geishecker (2012) provides a theoretical foundation (supported empirically) for expectations regarding the direction of such simultaneity bias. Geishecker (2012) derives the following expression to define the bias in a fixed effects estimate of the effect of economic insecurity on current wellbeing:

(1) $$Bias_{\delta }=\frac{\beta_{u}}{1-\beta_{u}\delta }\frac{Var(\varepsilon )}{Var(F)}$$

where δ is the effect of insecurity on current wellbeing, β_u_ is the effect of current wellbeing on insecurity, ε is the idiosyncratic error in the equation estimating current wellbeing, and F is perceived insecurity.

As the ratio of two variances is strictly positive for all informative models, Var(F)≠0, the second term in Eq. (1) affects only the size of the bias, not the direction. Geishecker (2012) focuses on the direction of bias, defined by the first term in Eq. (1), no predictions are made regarding the size of the bias.

From previous empirical results (Clark et al., 2010, Rohde et al., 2016, Watson, 2015) we believe that δ<0. That is that perceived insecurity lowers current wellbeing. Furthermore, the conceptualisation of insecurity defined in Geishecker (2012) shows that insecurity increases as the difference between current wellbeing less the expected out-of-job wellbeing becomes positive. That is, there is a loss to wellbeing of reverting to the outside option. Therefore, current wellbeing enters the insecurity equation positively (β_u_>0), and the utility of the outside option enters negatively. Evidence of this relationship can be observed in Green (2011) where employability is shown to moderate the effect of job insecurity on mental health, since employability increases wellbeing in the outside option and reduces the difference compared to current wellbeing. Hence, in all cases where β_u_δ≠1 the bias in the effect of insecurity on current wellbeing is positive. Given that δ<0, this indicates that estimates which do no control for simultaneity will underestimate the detrimental effect of economic insecurity on wellbeing/mental health.

We expand on the extant literature by analysing the effect of economic insecurity on mental health within a causal model which allows for variation between sources of insecurity, which may be either objective or subjective in nature. Furthermore, our analysis controls for both past and future unemployment outcomes, and allows for the experience of insecurity to differ across genders. Lastly, the robustness of our findings are enhanced by testing the theoretical predictions of Geishecker (2012) regarding the direction of potential simultaneity bias within the insecurity-mental health relationship. This strategy enables a fixed effects model to be used to identify a lower bound for the negative effect of economic insecurity on mental health within the working-age population of the UK.

## 3 Methodology

### 3.1 Data and selection of sample

The data used within the analysis comes from the BHPS (Institute for Social and Economic Research, 2010). All eighteen waves of available data are used to build the dataset. We measure the proportion of time spent unemployed within the last three years, and form a dummy variable indicating if the individual has a spell of unemployment within the next year. The need to include these three lags and one lead restricts the sample to the period covering 1993–2007. It was not possible to extend the sample beyond this point due to a discontinuity in the way that income is measured in the BHPS and the Understanding Society study, which replaced the BHPS after 2008. The need for a minimum of four consecutive periods of data for an individual also reduced our potential sample by approximately half. The focus for the analysis is working-age (16–64) sample members. Descriptive statistics for all variables are contained within Table A.1.

A number of additional sample restrictions are imposed in order to allow for a distinct effect of economic insecurity on health to be identified. Firstly, sample members must be part of the ‘primary’ workforce – that is employed on a permanent full-time contract. This restriction is imposed to address the possibility of individuals voluntarily or knowingly selecting into insecure employment, although it must be recognised that individuals may select into secure employment. However, the purpose of this restriction is to form a sample of employees who could reasonable expect security in their employment relationship, so selection into secure employment is a lesser concern. A further restriction is that the sample is limited to individuals either without a partner or whose partner is not suffering from either work-related or financial insecurity. Although intra-household transmission of economic insecurity is an interesting aspect that has not been thoroughly investigated to date, the aim of the current analysis is to accurately identify the health effect of economic insecurity on the immediate sufferer of insecurity. As a result of these sample restrictions 224 male and 949 female respondents are lost from the potential sample. By focusing on a section of the workforce which would traditionally be considered secure, it is likely that we underestimate the incidence of economic insecurity within the population and any associated mental health effects.

### 3.2 Dependent variable

The dependent variable in the analysis comes from responses to the GHQ-12. The questions cover aspects of mental functioning and emotional difficulties (see Appendix 2 for the question wording). Responses to the individual questions within the GHQ-12 are scored on a scale ranging from 0 (substantial decrease in symptoms compared to usual) to 3 (substantial increase in symptoms compared to usual). The twelve scores are then summed to form a Likert scale from 0 to 36 capturing a single dimension of mental wellbeing. In keeping with the relevant literature, this score has been reversed such that the scale is increasing in mental wellbeing. Additionally, the scale has been standardised to allow coefficients to be interpreted as standard deviations from the mean.

#### 3.2.1 Objective measurement of economic insecurity

In this paper we construct and use the Economic Security Index for Great Britain (ESIGB) which is obtained by applying some methodological changes to the original US-based ESI method proposed by Hacker et al. (2014). Departures from the original ESI methodology result from data issues and a focus on forming the index using a single data source.

As in Hacker et al. (2014) the final index is formed by finding the proportion of the population suffering a qualifying income loss in each period *t*:

(2) $$ESIGB_{t}=\frac{\sum_{i=1}^{n_{t}}L_{it}^{GB}}{n_{t}}$$

Individuals (*i*) within a household that has suffered a qualifying income loss meet both conditions within Eq. (3). Hacker et al. (2014) include a third condition that individuals must not be transitioning in to retirement between periods. Since our sample members are all employed, we have omitted this from Eq. (3). A qualifying loss is defined as:

(3) $$L_{it}^{GB}=\{\begin{array}{cc} 1 & if\;\left(\frac{y_{it}-D_{it}}{e_{it}}<\left(\frac{3}{4}\right)\frac{y_{it-1}-D_{it-1}}{e_{it-1}}\right)\cap (W_{it}<W_{it}^{*})\\ 0 & otherwise \end{array}$$

where *y* is real gross household income; *D* is real non-discretionary household costs (mortgage or rent payments); *e* is the modified OECD household equivalence scale (Hagenaars et al., 1994); *W* is the real household liquid financial wealth; and W* is the total income loss incurred over the median time for income to recover to pre-loss levels.

In order to obtain a measure of household wealth from the constituent personal wealth measures, the bounding approach within Banks et al., (2003) was used. The lower bound assumes that any jointly held asset is split evenly amongst the adults within the household. Amongst individuals within households that are identified as suffering from economic insecurity according to the ESIGB classification, the median level of liquid household wealth is £10, while the 75th percentile is only £1143. The median level of household wealth is higher within the full sample at £787. The 75th percentile amongst the full sample is much higher at £7402. This suggests that from a methodological perspective household wealth is relatively unimportant within the ESIGB since the majority of households at risk of economic insecurity have low wealth holdings. For this reason a detailed discussion of how the wealth components of the ESIGB (*W* and W*) is not given here, but is available within Appendix 3.

In addition to the complete ESIGB a simpler indicator identifying individuals suffering from a qualifying loss of household income, as defined by the first term in (3) above, is included in the main analysis. To be clear, this variable only includes household income (*y*) less non-discretionary spending (*D*), and is adjusted for household composition (*e*). The wealth (*W*) term is excluded. This has been done to ensure consistency of the sample for years in which no wealth data is available. Due to the low wealth levels observed, the rate of qualifying income drops provides a very close approximation to the full ESIGB.

#### 3.2.2 Subjective measurement of economic insecurity

Two subjective measures of economic insecurity are included in the analysis. The first is formed using individuals' expectations regarding their financial situation over the next twelve months. Individuals indicating that they expect their financial situation to worsen (originally on a 3-point scale) are coded as 1, while others are coded as zero. This subjective measure is called financial insecurity.

The second subjective measure comes from a question capturing individuals’ levels of satisfaction with their current job security.^2^ Responses are given on a 7-point scale. These have been recoded into a binary variable such that those expressing any dissatisfaction with their current level of job security are coded as 1. This subset is called work-related economic insecurity.

Work-related economic insecurity is distinct from conceptualisations of job insecurity which involve only the risk of involuntary employment termination. The key distinction being that information other than the probability of job loss, such as the financial and non-financial consequences of employment volatility, is assumed to be contained within responses. Such a conceptualisation is broadly similar to the cognitive and affective insecurity distinction presented by Anderson and Pontusson (2007).

**Fig. 1:**
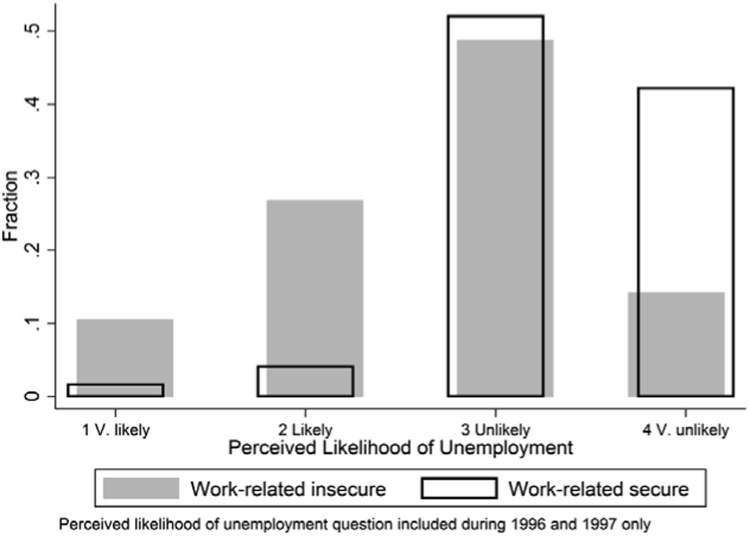
Comparison of Unemployment Expectations and Work-related Insecurity Classification (1996 and 1997 only).

To investigate the validity of this assumption, we compare responses on two related questions. Within two waves of the BHPS respondents were asked “In the next twelve months how likely do you think it is that you will become unemployed?” as an additional question. Responses were given on a four-point scale ranging from “1 Very likely” to “4 Very Unlikely”. These responses are presented in Fig. 1 along with the proportion of respondents within each category that were classified as suffering (or not) from work-related economic insecurity, as defined above.

The key feature of Fig. 1 is that a large group of individuals express dissatisfaction with current job security despite perceiving a low likelihood of unemployment. These individuals are contained within the third and fourth shaded columns. This suggests responses to the question regarding satisfaction with current job security, and, as such, our work-related insecurity variable, likely involves more than just an assessment of becoming unemployed. It should be noted that an employment relationship can be involuntarily terminated without unemployment necessarily occurring if another job can be found quickly. Although such job loss is still likely to be costly since replacement jobs could be expected to be inferior. Therefore, Fig. 1 does not capture the full complexity of the relationship between insecurity and employment volatility, but it is presented to highlight a conceptual distinction between unemployment perceptions and work-related insecurity which may influence comparisons with studies which use an alternative question wording.

### 3.3 Model specification

The specification used to identify a lower bound for the effects of economic insecurity on mental health takes the form:

(4) $$H_{it}=\beta_{0}I_{it}+\beta_{1}P_{it}+\beta_{2}F_{it}+X_{it}^{\prime }\gamma +\alpha_{i}+\eta_{t}+\varepsilon_{it}$$

where *H*_*it*_ is the standardised GHQ-12 score for individual *i* at time *t*, *I* is the dummy variable indicating exposure to economic insecurity (work-related, financial, qualifying income loss, or ESIGB), *P* is a continuous variable capturing the proportion of time spent unemployed in the past 3 years, and *F* is a dummy variable which equals one if the individual had a spell of unemployment (regardless of duration) in the next 12 months. *X* is a vector of standard controls (percentiles of equivalised household income, existing medical condition dummy, education dummies, age bands, marital status dummies, number of children, industry of employment dummies, log of hours worked, and employer size dummies). The individual-specific intercept is given by α, η is the time dummy, and ε represents the idiosyncratic error. All standard errors are clustered at the individual level such that they are robust to arbitrary heteroscedasticity and within-subject autocorrelation.

The model above is first estimated using the fixed effects (FE) estimator. This approach requires that the dependent variable is cardinal in nature, which is evidently not the case when using the GHQ-12 score. However, such an approach is common within the relevant literature (Clark et al., 2010, Rohde et al., 2016, Watson, 2015), and a benefit of this strategy is that results are readily comparable to those produced by the instrumental variables (IV) estimation.^3^

A valid comparison between the FE and IV results is vital if a lower bound for the effect of economic insecurity on mental health is to be identified. The FE results are assumed to suffer from simultaneity bias which results in the coefficient being smaller (in absolute terms) than would otherwise be found. IV results, which do not suffer from simultaneity bias if sufficiently strong instruments are employed, should exhibit larger negative effects of insecurity on mental health. Thus, the FE results can be considered to be a conservative estimate of the ‘true’ effect size.

To enable IV estimation, variations in levels of insecurity within individuals’ reference groups are used. These instruments are constructed at the 2-digit occupation (17 categories), 2-digit industry (25 categories), and region (12 categories) levels. In each case the mean level of the relevant form of economic insecurity is used. For example, in an IV regression with financial insecurity as the dependent variable, all individuals within London will have the mean level of financial insecurity in London as one of the exogenous instruments. Two further instruments would be assigned based on the individual’s industry and occupation. A minimum sample size restriction is applied to form the instruments. In each case, the first percentile of category size is used as the cut-off. For every year the mean level of insecurity is calculated with each industry category having at least 35 observations, each occupation category having at least 30 observations, and each region category having at least 140 observations.

The rationale behind the instruments is that changes in mean insecurity at the region, industry, or occupational level will only impact on individuals’ mental health by altering expectations about their own employment or financial situation – i.e. individuals will form expectations by taking cues from their economic reference groups. Importantly, awareness of the mean levels of insecurity will not impact on individuals’ current mental wellbeing directly. Any effect will occur only by altering their economic insecurity – i.e. their level of concern for their future situation. Results from an ancillary regression are presented in Table A.2 where the instruments were shown to be both jointly and individually insignificant (p>0.1) in a regression on GHQ-12 score. These results suggest that the instruments are correctly excluded from the outcome equation of interest since we fail to reject the null hypotheses that aggregate insecurity has no direct effect on mental health.

## 4 Results and discussion

Sub-Section 4.1 offers a comparison of trends in the measures of economic insecurity, 4.2 gives the results of the FE regression, and Sub-Section 4.3 presents results from the IV regression.

### 4.1 Comparison of trends

**Fig. 2:**
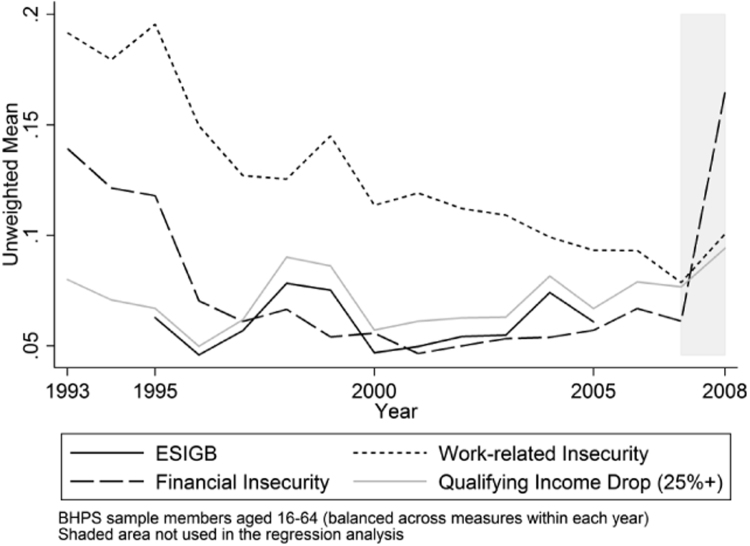
Mean Levels of ESIGB and Subjective Measures of Economic Insecurity.

Given the difficulty of capturing economic insecurity within a single measure it is worthwhile to examine a range of indicators. Fig. 2 illustrates the mean level in each of the measures of economic insecurity. It is apparent that all measures show a decline in the level of economic insecurity until around 2001. This decline coincides with an extended period of macroeconomic stability, with the national unemployment rate, which can considered to be the objective risk of unemployment, also declining. Similar results showing declining levels of economic insecurity have been found for Australia (Rohde et al., 2014), although in the US it appears that economic insecurity increased over the same period (Hacker et al., 2014).

Beyond 2001 the trends differ. The full ESIGB, qualifying income drops, and financial insecurity show a slight increase until 2007. The only measure that does not display any clear increase beyond 2001 is the measure of work-related insecurity. The trend in this measure declines steadily. However, in all periods within our sample (1993–2007) work-related insecurity affects the greatest proportion of the sample compared to the other insecurity measures.

Our sample period predates the Great Recession, which would be expected to alter the trends in economic insecurity within Fig. 2. However, the period during and following the Great Recession is also of interest. As such we present some roughly comparable figures within the shaded area of Fig. 2 and in Table 1.^4^

**Table 1:** Economic Insecurity in the UK since the Great Recession.

Period covering	2009–2011	2010–2012	2011–2013	2012–2014	2013–2015	2014–2016	2015–2017
Financial insecurity	16.13%	17.79%	19.91%	16.55%	14.52%	9.70%	9.83%
Unemployment likelihood	–	12.03%	–	9.83%	–	6.84%	–

Notes:

Unemployment likelihood, available every second year, asks respondents to rate the likelihood of involuntarily losing their job over the next 12 months.

The financial insecurity definition is identical to that used in the main analysis.

Sample periods differ to the BHPS and overlap multiple years.

A striking feature of the shaded area in Fig. 2 is the substantial increase in financial insecurity between 2007 and 2008. The proportion of individuals who expected their financial situation to worsen increased from around 5% to over 15%. Table 1 suggests that this rate was sustained, or even increased, until as recently as 2014. We do not observe an increase of this scale in the other available measures of economic insecurity, although it does appear that insecurity also increased in these measures following the Great Recession.

Due to the reasons outlined in Section 3.1. we are unable to include the period beyond 2007 within the current study. The analysis within the rest of the paper focuses on the period from 1993 to 2007.

### 4.2 Fixed effect regression

Table 2 gives the results from the FE regressions for each measure of economic insecurity. FE is used to control for confounding variables and individual heterogeneity. Both future orientated measures of economic insecurity (work and financial) result in a significant negative effect on mental health in males and females. For males, being dissatisfied with current levels of job security reduces mental wellbeing by 0.316 of a standard deviation from the mean. The equivalent figure for females is 0.171. Using Canadian data, Watson (2015) finds a negative effect on male mental health of 0.14 standard deviations from the mean, and 0.09 for females. For both genders the size of this effect is lower than those reported in Table 2 although the pattern of work-related insecurity affecting males to a greater extent is observed.

**Table 2:** The Effect of Economic Insecurity on Standardised GHQ-12 score (Fixed Effect Regression).

Dependent variable:	Male	Female	Male	Female	Male	Female	Male	Female
GHQ-12 score	Work-related	Work-related	Financial	Financial	Income	Income	ESIGB	ESIGB
Economic insecurity	−0.316***	−0.171***	−0.181***	−0.175***	−0.048	0.018	−0.069	0.007
	(0.033)	(0.043)	(0.036)	(0.055)	(0.034)	(0.052)	(0.044)	(0.063)
								
Unemployment experience	0.311	−0.243	0.328*	−0.270	0.337*	−0.260	0.339	−0.109
	(0.196)	(0.334)	(0.195)	(0.331)	(0.193)	(0.331)	(0.249)	(0.329)
								
Unemployment anticipation	−0.203**	0.007	−0.261***	−0.012	−0.264***	−0.035	−0.255***	0.005
	(0.079)	(0.157)	(0.080)	(0.153)	(0.080)	(0.155)	(0.093)	(0.172)
								
2nd income quintile	−0.024	0.033	−0.015	0.035	−0.026	0.038	−0.039	−0.055
	(0.031)	(0.047)	(0.031)	(0.047)	(0.032)	(0.047)	(0.039)	(0.052)
								
Middle income quintile	−0.029	0.014	−0.024	0.008	−0.038	0.015	−0.067	−0.053
	(0.037)	(0.055)	(0.038)	(0.055)	(0.039)	(0.055)	(0.047)	(0.063)
								
4th income quintile	−0.032	−0.023	−0.025	−0.026	−0.044	−0.019	−0.063	−0.112
	(0.044)	(0.058)	(0.044)	(0.058)	(0.045)	(0.060)	(0.053)	(0.072)
								
Top income quintile	0.043	−0.028	0.055	−0.032	0.029	−0.021	−0.013	−0.161*
	(0.055)	(0.071)	(0.055)	(0.071)	(0.057)	(0.073)	(0.068)	(0.087)
								
Existing medical condition	−0.124***	−0.146***	−0.124***	−0.145***	−0.124***	−0.145***	−0.098***	−0.135***
	(0.025)	(0.033)	(0.025)	(0.033)	(0.025)	(0.033)	(0.030)	(0.039)
								
Separated from partner	−0.571***	−0.283**	−0.554***	−0.279*	−0.553***	−0.290**	−0.558***	−0.159
	(0.144)	(0.143)	(0.145)	(0.143)	(0.145)	(0.142)	(0.175)	(0.183)
								
Other controls	Yes	Yes	Yes	Yes	Yes	Yes	Yes	Yes
Occupation, region, and year dummies	Yes	Yes	Yes	Yes	Yes	Yes	Yes	Yes
Observations	13186	7650	13186	7650	13186	7650	9845	5565
Individuals	2499	1690	2499	1690	2499	1690	2168	1375
R^2^	0.037	0.023	0.027	0.022	0.024	0.020	0.026	0.024

Notes:

Clustered standard errors in parentheses.

* p<0.10.

** *p*<0.05.

*** *p*<0.01.

Gender differences are not observed for the other insecurity variables. When an individual expects their financial situation will worsen within the next year this reduces mental health by roughly 0.18 of a standard deviation for both males and females. In line with Knabe and Rätzel (2011), there is no statistically significant (at the 5% significance level) effect from past unemployment when current work-related insecurity is controlled for.

Table 2 shows that subjective elements of economic insecurity have a stronger negative impact on mental health than the objective measures used. There are no statistically significant effects associated with either experiencing a 25% or more decrease in household income or the full ESIGB index. For both males and females, it appears that exposure to perceived risk is more harmful than the realisation of risk. This is an important point since individuals may suffer a negative effect on mental health regardless of the perception of risk being either justified or realised. Therefore, these results suggest that the negative effects of economic insecurity may be hidden from conventional measurement by objective indicators.

The analysis of Rohde et al. (2016) enables some comparison across a range of economic insecurity measures affecting mental health. Rohde et al. (2016) find that financial insecurity is slightly more damaging to mental health than work-related insecurity. This differs from the results in Table 2 where work-related insecurity is clearly more damaging than financial insecurity in males, although Rohde et al. (2016) do not separate their sample by gender. It is particularly informative that Rohde et al. (2016) also find no statistically significant effect of a drop in household income of 25% or more. Rohde et al. (2016) impose an additional condition that income during the period in which the loss is incurred must be below the household-specific mean income over the entire sample period. The results in the last four columns of Table 2, along with those in Rohde et al. (2016), suggest that transitory income shocks have little impact on mental health. One reason for this may be that such shocks are the result of planned transitions. Alternatively, the psychological distress resulting from an unplanned, but expected, loss may occur prior to the event, and as such may be more likely to be captured by the financial insecurity variable.

As discussed in Section 3.2.1, liquid financial wealth holdings are often minimal amongst those suffering a qualifying income loss. This suggests that asset poverty may be a major risk factor associated with the ESIGB, since sufferers of economic insecurity are more likely to have little or no protection from household income volatility. Therefore, if such a loss was unexpected it is plausible that these individuals would target credit markets to meet any shortfall in income. Such a scenario could cause chronic psychological distress as individuals manage the resulting burden of debt over multiple periods.

To provide some context as to the relative size of the effect of insecurity, it is useful to make a comparison with other statistically significant variables. Suffering from one or more existing health problems has a negative effect on mental health of approximately 0.12 and 0.15 for males and females, respectively. Therefore, at the individual level either work-related or financial insecurity is more damaging to mental health than suffering from an existing health problem. A further comparison can be made to a major life event - separating from a partner. For males and females, separating from a partner has a larger negative effect on mental health than all forms of insecurity. The negative impact for males is approximately 0.56 standard deviations from the mean, and for females around 0.28.

It is also useful to employ the MDD concept to assess the magnitude of coefficients. The MDD is the threshold level of change in an outcome that an individual would perceive as either beneficial or harmful and which would lead to the individual altering how they manage the issue (Schunemann et al., 2005). Across many studies this threshold has been observed to be between 0.3 and 0.5 standard deviations from the mean (Norman et al., 2004). Therefore, this level can be considered to be a valid approximation in the absence of clinically observed values. By this measure the gender differences become more significant for work-related insecurity since for males the coefficient is above the MDD threshold, while for females it is below this threshold. Consequently, male sufferers may change their behaviour in response to insecurity. This could include seeking clinical treatment or altering other behaviours, for example, taking a period of time off work. It is also possible that a change in behaviour aimed at coping with psychological distress may produce further negative health outcomes, such as obesity and hypertension (Ferrie et al., 1995, Ferrie et al., 2002, Niedzwiedz et al., 2017, Rohde et al., 2017b).

To investigate if exposure to work-related economic insecurity did change behaviour we conducted a series of t-tests. We found a statistically significant difference in the number of hours worked by men who had suffered from work-related economic insecurity in the current (P<0.01) or previous (P<0.01) period compared with those who had not suffered insecurity. The insecure worked fewer hours. These differences were not found in the female sample. Furthermore, a higher incidence of smokers was observed amongst men who had suffered from work-related insecurity in the previous period (P<0.05). This difference was not observed in the period within which insecurity was suffered. Statistically significant differences in the incidence of smoking were found in both periods for the female sample (P<0.01). Although not unequivocal, these tests provide some support that work-related economic insecurity is associated with changes in behaviour which were more pronounced above the MDD threshold.

Pertinent to the analysis is the test of a “breadwinner hypothesis” conducted by Watson (2015). An alternative test of this “breadwinner hypothesis” was carried out in the current analysis by limiting the sample to only those identified within the BHPS as being legally or financially responsible for the household (2,159 males and 1,009 females). As with Watson (2015), this resulted in the coefficient for work-related economic insecurity becoming insignificant for females at the 5% significance level (p=0.068), while the equivalent coefficient for males became larger (β=−0.330) and remains statistically significant (p<0.01). Additional checks showed little difference in the proportion of household income being provided by male and female household heads. As such, it may not be the “breadwinner” role in itself that causes the gender differences that are observed in many studies of job insecurity, although it cannot be ruled out that the responsibilities of this role impact differently on males and females.

The results in this analysis support the suggestion that the source of this difference between genders involves more than financial aspects. Clark (2003) and Strandh et al., (2013) show that differing societal expectations regarding labour force participation of males and females can alter the negative impact on wellbeing resulting from unemployment. It may also be the case that such societal expectations influence the extent of psychological distress resulting from a fear of unemployment.

One gender difference shown in Table 2 which contradicts existing literature is that anticipation of unemployment has a statistically significant negative effect on males, but no effect on females. This result is apparent across all measures of economic insecurity that are used within the analysis. Conversely, Clark and Georgellis (2013) show a negative impact on GHQ score for both males and females prior to unemployment being experienced. This effect is larger for females. It should be noted, however, that the specifications in Table 2 differ from Clark and Georgellis (2013). In particular, the inclusion of current work-related economic insecurity would be expected to capture a similar effect to anticipation of unemployment, since work-related insecurity includes a cognitive assessment of the likelihood of suffering involuntary termination of employment.

Additional regressions interacting the economic insecurity variables with income, unemployment experience, and unemployment outcomes within the next 12 months were performed. Only one of these individual interactions was statistically significant at the 5% level (financial insecurity and unemployment experience in females), and in every case the interactions were jointly insignificant. Therefore, the decision was made to exclude these interactions from the main results. However, the lack of statistically significant interactions may still be informative. Firstly this shows that the negative effect of work-related and financial insecurity is experienced regardless of future unemployment outcomes. More importantly, the negative effects of work-related and financial economic insecurity on mental health do not appear to change depending on the sufferer’s position within the household-income distribution. Such a finding is consistent with results using Australian data reported by Rohde et al. (2017a) which showed that the sensitivity of health to insecurity varied little with increased income. Thus, suggesting that threats to an individual’s relative status, rather than a risk of poverty, is the principal factor in the insecurity and health relationship.

### 4.3 Instrumental variables regression

Table 3 reports the results of the IV estimation. The purpose of this stage of analysis is to test the hypothesis, based on Geishecker (2012), that the coefficients on the economic insecurity variables from the FE analysis (Table 2) underestimate the effects of economic insecurity on mental health due to simultaneity bias. This prediction can be readily applied to work-related economic insecurity. In the case of financial insecurity this is less clear as the source of the insecurity is not known, although this could conceivably result from an expectation of employment volatility. The objective insecurity measures are based on the experience of past events, and there is no reason to believe that current mental health will affect past events. Hence, simultaneity bias was neither suspected nor supported in these cases and the results are not reported in Table 3.

**Table 3:** The Effect of Economic Insecurity on Standardised GHQ-12 score (Instrumental Variables Regression).

Dependent variable:	Male	Female	Male	Female
Standardised GHQ-12 score	Work-related	Work-related	Financial	Financial
Economic insecurity	−0.890***	−0.012	−0.224	0.572
	(0.307)	(0.403)	(0.410)	(0.545)
				
Unemployment experience	0.256	−0.258	0.325*	−0.222
	(0.209)	(0.331)	(0.196)	(0.335)
				
Unemployment anticipation	−0.093	−0.032	−0.260***	−0.108
	(0.102)	(0.186)	(0.080)	(0.175)
				
2nd income quintile	−0.038	0.034	−0.015	0.032
	(0.033)	(0.047)	(0.031)	(0.048)
				
Middle income quintile	−0.037	0.009	−0.024	0.012
	(0.038)	(0.056)	(0.038)	(0.056)
				
4th income quintile	−0.043	−0.027	−0.024	−0.030
	(0.044)	(0.058)	(0.044)	(0.058)
				
Top income quintile	0.025	-0.030	0.055	−0.022
	(0.056)	(0.071)	(0.056)	(0.073)
				
Existing medical condition	−0.124***	−0.145***	−0.123***	−0.144***
	(0.026)	(0.033)	(0.025)	(0.033)
				
Separated from partner	−0.597***	−0.288**	−0.554***	−0.320**
	(0.145)	(0.142)	(0.144)	(0.141)
				
Other controls	Yes	Yes	Yes	Yes
Occupation, region, and year dummies	Yes	Yes	Yes	Yes
Observations	13186	7650	13186	7650
Individuals	2499	1690	2499	1690
Hansen J Overidentification stat	0.30	3.64	1.56	0.72
Overidentification p-value	0.86	0.16	0.46	0.70
LM Underidentification test stat	68.98	35.11	51.59	30.02
Underidentification p-value	7.07e-15	1.16e-7	3.65e-11	1.37e-6
K-P Weak Identification F stat	24.32	12.69	18.50	10.15

Notes:

Clustered standard errors in parentheses.

Instruments are mean insecurity at the industry, region, and occupation level.

* p<0.10.

** p<0.05.

*** p<0.01

For males, the hypothesis that the FE results underestimate the negative effects of work-related economic insecurity on mental health is supported. Diagnostics tests indicate that the instruments are sufficiently strong (Kleibergen-Paap F=24.32) to identify a coherent set of parameters (Parente & Santos Silva, 2012). Therefore, it is likely that this relationship does suffer from simultaneity bias. The coefficient is almost three times larger in the IV regression compared to the FE results, which is roughly equivalent to the bias found by Geishecker (2012) using a sample which pooled the genders. Furthermore, insecurity becomes the largest negative factor in the model and the anticipation of unemployment variable is no longer statistically significant, although we fail to reject the null hypothesis that this coefficient equals the value found in the FE regression (P>.1). It is not possible to make direct comparison of the bias size with other studies which do not control for potential simultaneity bias. However, it could be assumed that these studies underestimate the size of the negative effect.

The results for financial insecurity in males are less conclusive. Although the absolute value of the coefficient is larger in the IV model compared to the FE model, the coefficient is no longer statistically significant. This could be interpreted as there being no simultaneity bias. Such a finding would suggest that the sources of insecurity are not common to the work-related insecurity measure and that the FE result provide evidence of a causal relationship.

For females, the results appear to be affected by weak instruments, as reflected in the Kleibergen-Paap statistic, and so the coefficients and standard errors may be unreliable. To understand why we find little evidence of simultaneity in the insecurity and mental health relationship for females it is worthwhile to consider the social norm effects of unemployment (Clark, 2003). As Geishecker (2012) illustrates (Eq. 1 above), simultaneity bias in the insecurity and mental health relationship increases as the wellbeing derived from current employment increases. Social norms may influence the current in-job wellbeing. If this is higher for males than females $\left(\beta_{u}^{Male}>\beta_{u}^{Female}\right)$, as found by Clark et al. (2010), then this implies that we could expect simultaneity bias to be more substantial within the FE regression for males.

A further implication of social norm theory is that the reference group effect may be weaker for females compared to males. Our identification strategy assumes that individuals will respond to insecurity within their reference group. However, given the result of Clark et al. (2010) that the social norm of employment differs between genders, then the extent to which males and females respond to variations in the reference group will also differ. It is possible that males are more responsive to variations in the reference group as a result of social norms, and so the instruments are more effective.

## 5 Conclusions

This paper has added to an emerging body of evidence showing the negative effects of economic insecurity on mental health. Work-related economic insecurity in males is clearly identified as the most substantial source of negative mental health effects. Furthermore, it appears that a key feature of economic insecurity is that the negative effects on mental health are experienced equally throughout the income distribution and may be experienced without any objective event occurring. Consequently, economic insecurity may result in a largely hidden welfare loss resulting from psychological distress that can affect any workforce member. Since work-related insecurity often had the highest rate of incidence within the population, it is clear that work-related economic insecurity represents a major issue for population health. A greater focus on those who remain in employment, but suffer from stress and anxiety relating to the continuation of that employment, is required in order to address and understand this issue.

Theoretical predictions from Geishecker (2012) regarding the direction of the simultaneity bias were supported in the case of male employees. Therefore, the fixed effect regression results can be considered as a lower bound for the negative impact of economic insecurity on mental health. The results for females were less conclusive, although it is likely that the FE results are the most relevant. For working-age males, the estimated lower bound of the negative effect is 0.316 standard deviations from the mean, which is above the MDD threshold. This level of change in mental health would be perceived as harmful by the individual and may result in a change in health behaviours. Existing studies that do no control for simultaneity are likely to underestimate the negative effect of economic insecurity on male mental health. As a result of this underestimation researchers may erroneously interpret their results to be below the MDD threshold, implying that sufferers of economic insecurity may not alter their behaviour or perceive the insecurity to be harmful. Such an interpretation would understate the need for policy interventions to address the effects of economic insecurity on population health and productivity.

The focus of this paper has been a contemporaneous relationship between economic insecurity and mental health. Recent evidence (Niedzwiedz et al., 2017, Rohde et al., 2017b, Watson and Osberg, 2017) has shown that repeated exposure to economic insecurity can be more damaging to health than transitory spells. No attempt has been made to distinguish between transitory and chronic economic insecurity in the current study, and as such our results may be capturing a mixture of both. In addition, the Great Recession may have fundamentally changed labour market trends and the institutions that mitigate insecurity. Future research will attempt to investigate these issues further.

From a public policy perspective, the results suggest that the substantial costs associated with the treatment of mental health issues may be reduced with policies that target working-age males in full-time employment. This is a group that receives less focus than the more obviously vulnerable, such as the unemployed or those living in poverty. Further policy intervention at the employer level may also limit costs associated with absenteeism, reduced productivity, and higher staff turnover due to economic insecurity. To be most effective, such policies may also be targeted at particular sections of the workforce.

The analysis in this paper encourages investigations of how labour market institutions, employer characteristics, and management practices mitigate the negative impact of economic insecurity on mental health.

## Figures and Tables

**Table A.1:** Descriptive statistics.

	All (n=20836)	Male (n=13186)	Female (n=7650)
GHQ-12 score	25.393 (4.856)	25.811 (4.496)	24.672 (5.344)
Work-related economic insecurity	0.121 (0.326)	0.128 (0.334)	0.108 (0.311)
Financial insecurity	0.066 (0.248)	0.067 (0.25)	0.064 (0.246)
Qualifying income drop (25% or more)	0.070 (0.256)	0.071 (0.257)	0.068 (0.252)
			
ESIGB	0.060 (0.238)	0.060 (0.238)	0.059 (0.236)
	(n=15864)	(n=10085)	(n=5779)
Unemployment experience	0.009 (0.050)	0.010 (0.056)	0.006 (0.038)
Unemployment anticipation	0.012 (0.110)	0.014 (0.116)	0.010 (0.097)
Involuntary termination	0.020 (0.138)	0.022 (0.148)	0.015 (0.12)
No qualifications	0.139 (0.346)	0.152 (0.359)	0.116 (0.32)
Lower secondary	0.315 (0.464)	0.293 (0.455)	0.352 (0.478)
Upper secondary	0.234 (0.423)	0.247 (0.432)	0.211 (0.408)
Higher education	0.313 (0.464)	0.308 (0.462)	0.321 (0.467)
Bottom income quintile	0.201 (0.401)	0.223 (0.416)	0.163 (0.37)
2nd income quintile	0.201 (0.401)	0.208 (0.406)	0.190 (0.392)
Middle income quintile	0.202 (0.401)	0.202 (0.401)	0.202 (0.402)
4th income quintile	0.202 (0.401)	0.192 (0.394)	0.219 (0.414)
Top income quintile	0.194 (0.395)	0.176 (0.381)	0.225 (0.418)
Under 35	0.292 (0.455)	0.272 (0.445)	0.328 (0.470)
Age 35–44	0.356 (0.479)	0.366 (0.482)	0.339 (0.473)
Age 44–64	0.351 (0.477)	0.362 (0.481)	0.333 (0.471)
Married	0.647 (0.478)	0.705 (0.456)	0.548 (0.498)
Living as a couple	0.136 (0.343)	0.131 (0.338)	0.144 (0.351)
Widowed	0.011 (0.105)	0.005 (0.073)	0.021 (0.144)
Divorced	0.073 (0.261)	0.046 (0.208)	0.121 (0.327)
Separated	0.020 (0.141)	0.014 (0.118)	0.031 (0.173)
Never married	0.112 (0.316)	0.099 (0.299)	0.134 (0.341)
Number of children	0.746 (0.984)	0.871 (1.036)	0.532 (0.846)
Existing health problem	0.490 (0.500)	0.462 (0.499)	0.537 (0.499)
Log of hours worked	3.639 (0.139)	3.671 (0.129)	3.585 (0.138)
Less than 25 employees	0.250 (0.433)	0.238 (0.426)	0.27 (0.444)
25–99 employees	0.273 (0.445)	0.258 (0.438)	0.298 (0.457)
100–499 employees	0.276 (0.447)	0.298 (0.457)	0.237 (0.426)
500+ employees	0.202 (0.401)	0.206 (0.404)	0.194 (0.396)

Notes:

Standard deviation in brackets.

ESIGB can only be constructed for a period (1995–2005) shorter than the main sample due to the unavailability of data on household wealth.

**Table A.2:** The Effect of Economic Insecurity on Standardised GHQ-12 Score including Exogenous Instruments (Fixed Effect Regression).

Dependent variable:	Male	Female	Male	Female
Standardised GHQ-12 score	Work-related	Work-related	Financial	Financial
Economic insecurity	−0.311***	−0.173***	−0.181***	−0.180***
	(0.033)	(0.043)	(0.036)	(0.054)
				
Occupation-level insecurity	−0.231	−0.535	0.235	0.779
	(0.284)	(0.432)	(0.402)	(0.569)
				
Industry-level insecurity	−0.562	0.400	0.146	0.0755
	(0.375)	(0.495)	(0.516)	(0.677)
				
Region-level insecurity	−0.427	0.832	−0.614	0.755
	(0.514)	(0.685)	(0.612)	(0.837)
				
Unemployment experience	0.310	−0.239	0.329*	−0.253
	(0.195)	(0.332)	(0.194)	(0.330)
				
Unemployment anticipation	−0.199**	0.00168	−0.262***	−0.0165
	(0.079)	(0.156)	(0.080)	(0.152)
				
2nd income quintile	−0.0240	0.0331	−0.0151	0.0339
	(0.031)	(0.047)	(0.031)	(0.047)
				
Middle income quintile	−0.0290	0.0130	−0.0239	0.00727
	(0.037)	(0.055)	(0.037)	(0.055)
				
4th income quintile	−0.0319	−0.0241	−0.0248	−0.0281
	(0.043)	(0.058)	(0.044)	(0.058)
				
Top income quintile	0.0412	−0.0288	0.0535	−0.0351
	(0.054)	(0.071)	(0.055)	(0.071)
				
Existing medical condition	−0.124***	−0.147***	−0.123***	−0.145***
	(0.025)	(0.032)	(0.025)	(0.032)
				
Other controls	Yes	Yes	Yes	Yes
Occupation dummies	Yes	Yes	Yes	Yes
Region dummies	Yes	Yes	Yes	Yes
Year dummies	Yes	Yes	Yes	Yes
Observations	13186	7650	13186	7650
Individuals	2499	1690	2499	1690
R^2^	0.038	0.024	0.027	0.023

Notes:

Clustered standard errors in parentheses.

Other controls include education, age, marital status, number of children, industry of employment, hours worked, employer size.

Instruments are mean insecurity at the industry, region, and occupation level.

* p<0.10.

** p<0.05.

*** p<0.01.

## Appendix 1. Sample characteristics

See Table A.1 here.

## Appendix 2 GHQ-12 questions

Here are some questions regarding the way you have been feeling over the last few weeks. For each question please tick the box next to the answer that best describes the way you have felt.

Have you recently….

a) been able to concentrate on whatever you’re doing?
b) lost much sleep over worry?
c) felt that you were playing a useful part in things?
d) felt capable of making decisions about things?
e) felt constantly under strain?
f) felt you couldn’t overcome your difficulties?
g) been able to enjoy your normal day-to-day activities?
h) been able to face up to problems?
i) been feeling unhappy or depressed?
j) been losing confidence in yourself?
k) been thinking of yourself as a worthless person?
l) been feeling reasonably happy, all things considered?

## Appendix 3 Measurement of wealth components within the ESIGB

The liquid financial wealth measure (*W*) is calculated using modules of questions regarding personal savings conducted in 1995, 2000, and 2005. Prior to 1995 and after 2005, it is not possible to estimate household wealth and as result the ESIGB is calculated for the period 1995 to 2005 only. Liquid financial wealth is defined as being financial assets that can be readily accessed. The available data from the BHPS limits this to amounts held in savings accounts and investments. To enable calculation of the ESIGB between years when the wealth module was asked, it was assumed that changes between periods occurred linearly at the personal level. The results within the Section 4 show that the level of the ESIGB does not differ greatly for years where information on wealth was available to years where linear interpolation is used.

The calculation of *W** involves a number of steps. Initially, the number of years taken for household income to return to pre-loss levels is calculated for all individuals suffering a qualifying income loss (truncated at 6 years). These values are then used to calculate the median time for household income to recover within groups based on age when the qualifying loss occurred (18–34, 35–44, 45–64, 65+), household income quintiles prior to the loss, and tertiles of the qualifying loss (50%, 35–50%, and 25–35%). We assume that the monetary value of the qualifying loss occurs in each period until household income fully recovers. Therefore, the qualifying income loss in the first period is multiplied by the median income recovery time to form *W**.

## Appendix 4. Excludability of Instruments from the outcome of interest

See Table A.2 here.

## Footnotes

1 This contrasts with the broader research on the effect of aggregate economic shocks and overall health. Some papers (Ruhm, 2000, Tekin et al., 2017) find that economic shocks can generate better health, although negative effects are found for mental health outcomes.

2 The BHPS question asks “How satisfied would you say you are with the job security in your present job?”.

3 Performed in Stata using XTIVREG2 (Schaffer, 2015).

4 The data prior to 2008 and after 2008 come from two different datasets – the BHPS and Understanding Society, respectively. Although there is some overlap in respondents and questions, the two datasets are not strictly comparable. Thus, while we focus on the BHPS in the regression analysis below, we have added the later data in Table 1 for illustrative purposes.
